# In Vitro Assessment of Pesticides Toxicity and Data Correlation with Pesticides Physicochemical Properties for Prediction of Toxicity in Gastrointestinal and Skin Contact Exposure

**DOI:** 10.3390/toxics10070378

**Published:** 2022-07-08

**Authors:** Amélia M. Silva, Carlos Martins-Gomes, Tânia L. Silva, Tiago E. Coutinho, Eliana B. Souto, Tatiana Andreani

**Affiliations:** 1 Department of Biology and Environment, School of Life Sciences and Environment, University of Trás-os-Montes e Alto Douro (UTAD), Quinta de Prados, 5001-801 Vila Real, Portugal; camgomes@utad.pt (C.M.-G.); tanialfs10@gmail.com (T.L.S.); tecoutinho@utad.pt (T.E.C.); 2 Center for Research and Technology of Agro-Environmental and Biological Sciences (CITAB-UTAD), Quinta de Prados, 5001-801 Vila Real, Portugal; tatiana.andreani@fc.up.pt; 3 Department of Pharmaceutical Technology, Faculty of Pharmacy, University of Porto, Rua de Jorge Viterbo Ferreira, 228, 4050-313 Porto, Portugal; ebsouto@ff.up.pt; 4 UCIBIO/REQUIMTE, Faculty of Pharmacy, University of Porto, 4050-313 Porto, Portugal; 5 GreenUPorto—Sustainable Agrifood Production Research Centre & Department of Biology, Faculty of Sciences of the University of Porto, Rua do Campo Alegre s/n, 4169-007 Porto, Portugal

**Keywords:** glyphosate, imidacloprid, imazalil, cytotoxicity, in vitro cell line models, water solubility, partition coefficient, toxicity prediction

## Abstract

In this work, three pesticides of different physicochemical properties, namely, glyphosate (herbicide), imidacloprid (insecticide) and imazalil (fungicide), were selected to assess their cytotoxicity against distinct cell models (Caco-2, HepG2, A431, HaCaT, SK-MEL-5 and RAW 264.7 cells) to mimic gastrointestinal and skin exposure with potential systemic effect. Cells were subjected to different concentrations of selected pesticides for 24 h or 48 h. Cell viability was assessed by Alamar Blue assay, morphological changes by bright-field microscopy and the IC_50_ values were calculated. Cytotoxic profiles were analysed using the physico-chemical parameters of the pesticides, namely: molecular weight, water solubility, the partition coefficient in the *n*-octanol/water (Log P_ow_) system, the topological polar surface area (TPSA), and number of hydrogen-bonds (donor/acceptor) and rotatable bonds. Results showed that glyphosate did not reduce cell viability (up to 1 mM), imidacloprid induced moderate toxicity (IC_50_ > 1 mM for Caco-2 cells while IC_50_ = 305.9 ± 22.4 μM for RAW 264.7 cells) and imazalil was highly cytotoxic (IC_50_ > 253.5 ± 3.37 for Caco-2 cells while IC50 = 31.3 ± 2.7 μM for RAW 264.7 cells) after 24 h exposure. Toxicity was time-dependent as IC_50_ values at 48 h exposure were lower, and decrease in cell viability was accompanied by changes in cell morphology. Pesticides toxicity was found to be directly proportional with their Log P_ow_, indicating that the affinity to a lipophilic environment such as the cell membranes governs their toxicity. Toxicity is inverse to pesticides TPSA, but lower TPSA favours membrane permeation. The lower toxicity against Caco-2 cells was attributed to the physiology and metabolism of cell barriers equipped with various ABC transporters. In conclusion, physicochemical factors such as Log P_ow_, TPSA and H-bond are likely to be directly correlated with pesticide-induced toxicity, thus being key factors to potentially predict the toxicity of other compounds.

## 1. Introduction

Nowadays, agricultural activities are highly dependent on pesticides use. It is commonly accepted that they play an important role in the development of agriculture by reducing the loss of products while improving the yield and food quality [1,2,3,4]. The term “pesticides” comprises a vast number of compounds, such as fungicides, herbicides, insecticides, molluscicides, nematicides, rodenticides, plant growth regulators and others, according to the pest target [2,5]; for example, herbicides, insecticides and fungicides are used worldwide to kill weeds, or unwanted plants, insects and fungi, respectively [1,5,6]. Undoubtedly, the use of pesticides has brought great benefits both in increasing the availability and quality of food and in supporting public health in general [7]. However, misuse or overuse of pesticides result in a vast range of negative impacts to the environment, to species diversity and to animal and human health, as is well documented in many toxicological studies [2,8]. It is also documented that the presence of pesticides in food products which is deemed to be in trace quantities represents low toxicity risk to consumers; however, the same might not be true to occupational populations that are exposed to high doses while mixing or applying the pesticides [2,8,9]. Thus, the main routes of exposure to pesticides are through skin and respiratory contact, mainly for the occupational population, and through the gastrointestinal tract (oral route), mainly for consumers [1,8], resulting in several adverse conditions, depending on the dose and exposure time, including cancer, neurotoxicity, pulmonotoxicity, endocrine disruption, metabolic toxicity and others [8]. Assessment of risk toxicity is not an easy issue, due to many factors, such as type of pesticide, exposure route, concentration of pesticide and duration of exposure. When using pesticide formulations the toxicity of the other formulation ingredients must also be considered, as pointed out by many authors [1,8,10,11].

**Figure 1:**
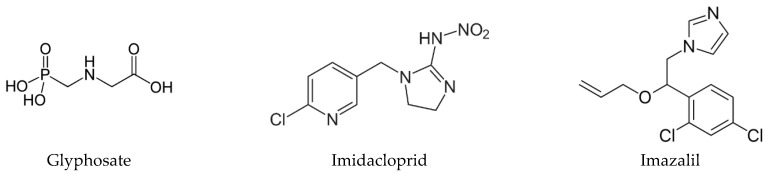
Chemical structure of glyphosate, imidacloprid and imazalil.

Thus, we aimed to study the cytotoxic effect of the neat active ingredient of three pesticides, an herbicide, an insecticide and a fungicide, and from these classes we choose glyphosate (GLY), imidacloprid (IMD) and imazalil (IMZ), respectively. This choice was ground on the fact that they have different chemical structures (Figure 1), different water solubility and are directed to different molecular targets. The herbicidal action of GLY (*N*-phosphonomethyl-glycine) is achieved by the inhibition of 5-enolypyruvylshikimate-3-phosphate synthase (EPSPS; EC 2.5.1.19) metabolic pathway; this enzyme is produced by and is present in plants, fungi and some microorganisms, but is not present in animals. EPSPS catalyses the transference of the enolpyruvyl group from phosphoenolpyruvate to shikimate-3-phosphate. Thus, GLY inhibits the biosynthesis of chorismate from shikimate, in a metabolic pathway deeply involved in the biosynthesis of essential metabolites, such as amino acids (e.g., phenylalanine, tyrosine or tryptophan) [1,12,13]. IMD is a neonicotinoid insecticide selective for the nicotinic acetylcholine receptor (nAChR) that exhibits higher affinity for insect nAChR than for vertebrate nAChR [14,15]. Although developed to target insect nAChR, several studies report adverse effects on vertebrate cell and in vivo models. This is concerning since high levels of IMD and of its metabolites have been detected in several food products, such as honey, fruits and vegetables [16]. IMZ, also known as enilconazole, is a broad-spectrum systemic fungicide used worldwide to prevent postharvest decay of fruits, mainly citrus fruit, vegetables and ornamentals [17]. IMZ targets the cytochrome P450-dependent sterol 14α-demethylase (Cyp51; EC 1.14.13.70) and blocks C14-demethylation of lanosterol, which is a precursor of ergosterol; it thus blocks ergosterol biosynthesis [18], a target that is specific to fungi.

In principle, these pesticides were designed with the aim of reaching specific targets, in particular aiming at a specific molecular site of the target species that is not present in the metabolic pathways of humans, to reduce the risk of toxicity to consumers. However, is the toxicity of pesticides essentially due to their interaction with their respective molecular targets or are the physicochemical properties inherent to the pesticide molecule the cause of non-specific toxicity?

Previous studies have analyzed the individual toxicity of these pesticides in a small number of animal cell models, but so far there is no report comparing the toxicity of GLY, IMD and IMZ in the same conditions. Caco-2 cells (human epithelial colorectal adenocarcinoma) were previously used as a model to assess the toxicity of these pesticides, but separately, with reported IC_50_ values of 17.2 mg/mL (~102 mM) for GLY [19] and 30 µg/mL (~100 µM) for IMZ [20], and cytotoxicity for Caco-2 cells exposed to IMD at concentrations above 0.25 µg/mL (~0.98 μM) [21]. The effect of GLY was assessed in HepG2 cells exposed to low concentrations, up to 3.5 µg/mL (i.e., 20.7 µM), and a slight increase in cell proliferation was reported after 4 h and 24 h exposure [22]. However, in relation to other cell lines, no studies were found on the cytotoxic effect of these pesticides, although there are some studies on their effect in animal models, as will be discussed later. Since the number of toxicity studies of these pesticides in other cellular models relevant to occupational exposure is limited, it is necessary to increase the knowledge about the cytotoxic effect of these pesticides.

Thus, the main objective of the current work was to assess the toxicity of three pesticides, glyphosate, imidacloprid and imazalil, an herbicide, a fungicide and a systemic insecticide, respectively, on six different cell lines, namely: Caco-2 (human epithelial colorectal adenocarcinoma), HepG2 (human hepatocellular carcinoma); A431 (human epidermoid carcinoma); HaCaT (human keratinocytes), SK-MEL-5 (human skin melanoma); and RAW 264.7 (Mouse macrophages). The choice of these cell lines was based on the fact that Caco-2 (colon) and HepG2 (liver) would mimic a gastrointestinal exposure route, the skin cell lines (A431, HaCaT and SK-MEL-5) would mimic a skin exposure route, and Raw 264.7 cells, a macrophage cell line, would mimic a systemic action. It was also an objective to compare the cytotoxicity of pesticides with their main physicochemical parameters, namely the water solubility, the partition coefficient in the *n*-octanol/water (Log P_ow_) system, the topological polar surface area and hydrogen-bonds, the pKa, in order to extrapolate to other cell types and to predict effects of chronic exposure.

## 2. Materials and Methods

### 2.1. Materials and Reagents

Glyphosate (PESTANAL^®^, analytical standard), Imidacloprid (PESTANAL^®^, analytical standard) and Imazalil (PESTANAL^®^, analytical standard) were purchased from Merck (Darmstadt, Germany). Versene, trypsin-EDTA, Dulbecco’s Modified Eagle Medium (DMEM), penicillin, streptomycin, sodium pyruvate, L-glutamine and foetal bovine serum (FBS) were obtained from Gibco (Alfagene, Invitrogen, Lisbon, Portugal). Alamar Blue^®^ was purchased from Invitrogen, Life-Technologies (Porto, Portugal).

### 2.2. Cytotoxicity Evaluation

#### 2.2.1. Cell Maintenance and Handling

To assess the cytotoxic activity of GLY, IMD and IMZ, six cell lines, namely Caco-2 (human epithelial colorectal adenocarcinoma; Cell Lines Service (CLS), Eppelheim, Germany), HepG2 (human hepatocellular carcinoma; ATCC, Rockville, USA), A431 (human epidermoid carcinoma; CLS, Eppelheim, Germany), HaCaT (human keratinocytes, CLS, Eppelheim, Germany) [23], SK-MEL-5 (human skin melanoma; CLS, Eppelheim, Germany), and RAW 264.7 (mouse macrophages, Abelson murine leukaemia virus-induced tumour; CLS, Eppelheim, Germany) cells, were used. All cell lines were cultured in complete culture media, composed of Dulbecco’s Modified Eagle Media (DMEM), supplemented with 1 mM L-glutamine, 10% (*v*/*v*) foetal bovine serum (FBS), and antibiotics (penicillin at 100 U/mL, and streptomycin at 100 μg/mL) and maintained in an incubator (5% CO_2_/95% air; 37 °C, controlled humidity). Cells were grown, in T25 culture flasks, to near confluence.

Near confluence, all cell lines, except RAW 264.7 cells, were subjected to enzymatic treatment (trypsin-EDTA) for detachment and subculture, as described by Andreani et al. [24] and by Silva et al. [25], which was stopped using complete culture medium (1:1, trypsin:culture media) as soon as cells were detached. RAW 264.7 cells were scratched off from the culture flasks using a cell scratcher (Orange Scientific; Braine-L’Alleud, Belgium), handling was performed, as described by Silva et al. [25]. Once detached from the flasks, cells were gently separated and re-suspended using a Pasteur pipette, counted using an automated cell counter (TC10™, BIORAD, Lisbon, Portugal). Then, cells were re-suspended in fresh culture media (at 5 × 10^4^ cells/mL) and seeded into 96-well microplates (100 µL/well) which were maintained in an incubator, for 48 h (to adhere and stabilize) before being used. For other details, see [24,25].

#### 2.2.2. Cell Viability/Cytotoxicity Assay

Alamar Blue^®^ (AB) assay was performed to assess the in vitro effect of the pesticides on cell viability. In summary, cells seeded into 96-well microplates were treated with various concentrations of the pesticides (GLY: 0–1 mM; IMD: 0–1 mM; IMZ: 0–0.3 mM). Test solutions were prepared in FBS-free culture media by diluting the appropriate volume of respective stock solution. A stock solution of GLY was prepared in water (at 40 mM), and stock solutions of IMD and IMZ were prepared in DMSO (at 20 mM). After 24 h or 48 h of exposure, the test solutions were removed and immediately replaced with Alamar Blue solution (100 µL/well; 10% (*v*/*v*) in FBS-free medium), followed by an additional 5 h incubation. Then, absorbance was read at 570 and 620 nm using a microplate reader (Multiskan EX; MTX Lab Systems, Inc., Bradenton, FL, USA), and the percentage of AB reduction was calculated according to manufacturers’ instructions, as described [24]. Cell viability was calculated and expressed as percentage of control cells (non-exposed cells) [24].

The concentrations needed to reduce cell viability by 50% (IC_50_) were calculated from three independent experiments (each one done in quadruplicate) [26].

### 2.3. Evaluation of Cell Morphology

At the end of each set of experiments, microscopy analysis of cell morphology was performed using an inverted microscope (Lan Optics, Labolan, Esparza, Spain), in bright field, and photos were acquired using a Kern ODC881 Microscope camera (Kern & Sohn GmbH, Balingen, Germany). Acquisition was performed using MicroscopeVIS 1.0 Image Software (Kern & Sohn GmbH, Germany).

### 2.4. Data and Statistical Analysis

Data was analysed using tolls of Microsoft Excel and graphs were composed using GraphPad Prism version 7 (GraphPad Software Inc., San Diego, CA, USA). For the comparison of the IC_50_ values and the cytotoxic activity, statistical analysis was performed using GraphPad Prism version 7, applying the analysis of variance (ANOVA) followed by Tukey’s multiple test (α = 0.05).

## 3. Results

### Pesticide-Induced In Vitro Cytotoxicity

In this work, we report the comparison of in vitro toxicity of GLY, IMD and IMZ in various cell lines, chosen as models of different contact routes, and selected due to their different tissues of origin, different morphologic characteristics, and metabolic differences. As the largest organ in the human body and a primary exposure site to pesticides, the evaluation of skin sensitivity is here addressed by the use of three human cell lines: HaCaT (normal keratinocytes), A431 (epidermoid carcinoma) and SK-MEL-5 (malignant melanoma). In addition to skin contact, accidental ingestion of pesticides, or the ingestion of contaminated products, is among the common exposure routes to pesticides, and thus a cell model of the intestinal tract (Caco-2; human colorectal adenocarcinoma) was included. Aiming also to analyze the toxicity of pesticides after intestinal absorption, HepG2 (human hepatic carcinoma) cells were used since the liver plays a critical role in the first pass effect of xenobiotic metabolism. The RAW 264.7 (mouse macrophages) cells were used to assess the systemic effect as macrophages are present in every tissue.

**Figure 2:**
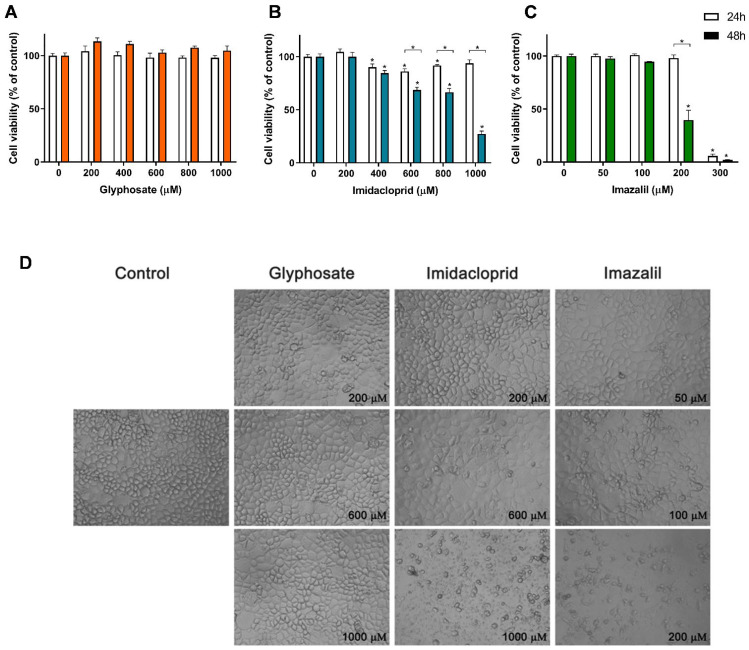
Effect of pesticides on Caco-2 cells viability and on cell morphology. Cells were exposed to GLY (**A**), IMD (**B**) and IMZ (**C**), for 24 h (white bars) or 48 h (filled bars), and the cell viability was assessed using Alamar Blue assay. Bright-field microscopy was used to assess morphological changes after 48 h exposure to the different pesticides, as indicated (**D**). Significant statistical differences between the control cells (non-exposed cells) and samples are denoted as “*” and between exposure times, for the same concentration, are denoted as “*” over a square bracket, when *p* < 0.05.

Figure 2 presents the cell viability results obtained for Caco-2 cells exposed to the three pesticides, namely GLY, IMD and IMZ (Figure 2A–C), as well as the effect of these pesticides on cell morphology (Figure 2D).

As observed in Figure 2, GLY did not induce toxicity in Caco-2 cells when applied at concentrations up to 1 mM (Figure 2A), where no significant reduction in cell viability was observed for either 24 h or 48 h exposure, at all tested concentrations. When confluent, this cell line presents a typical enterocyte differentiation, being commonly used in intestinal barrier assays, as they form an epithelial monolayer with cells tightly connected by intercellular junctions [27,28]. For these reasons, among all the tested cell lines, Caco-2 cells are ideal to analyze morphological changes induced by the pesticides. As observed in Figure 2D, Caco-2 cells exposed to GLY up to 1 mM presented control-like morphology which corroborates with the none cytotoxic effect observed in Figure 2A. On the other hand, Caco-2 cells exposed to 600 µM IMD presented some morphological changes, evidenced by a higher number of cells presenting large vacuoles, small round shaped cells (typical of cells being detached from the plate) and non-uniform shaped cells. At 1 mM of IMD, cell density is greatly reduced, mainly presenting cellular debris (Figure 2D). This is in line with the observed reduction in cell viability (Figure 2B), where concentrations ≥ 400 µM induced a significant reduction in cell viability. Caco-2 cells exposed to 600 µM and 1 mM of IMD for 48 h showed cell viability of 68.52% and 27.27% of control, respectively.

From dose-response curves identical to those presented in Figure 2, obtained for all cell lines exposed to the three pesticides for 24 h or 48 h, the IC_50_ values were calculated and are shown in Table 1. With IC_50_ values of 253.5 ± 3.4 µM and 186.5 ± 2.3 µM (24 h and 48 h, respectively), IMZ presented the highest toxicity against Caco-2 cells. At 300 µM, a cell viability reduction of 94.13% and 98.06% was observed for 24 h and 48 h exposure, respectively. Morphological analysis shows that at 100 µM IMZ induced significant changes in cell morphology, and that 200 µM IMZ (cell viability at 48 h exposure: 39.70%) clearly enhanced the morphological changes, with a significant reduction in cell density and an increase in cellular debris (Figure 2D). IMZ was, in fact, the most toxic pesticide tested in all cell lines evaluated, with IC_50′_s ranging between 7.21 ± 4.5 µM (RAW 264.7; 48 h exposure) and 253.5 ± 3.37 µM (Caco-2; 24 h exposure).

However, it was interesting to note that the intestinal epithelium cell line (Caco-2) presented the lowest sensitivity to all the tested pesticides, with the highest IC_50_ values obtained in all tested conditions.

**Figure 3:**
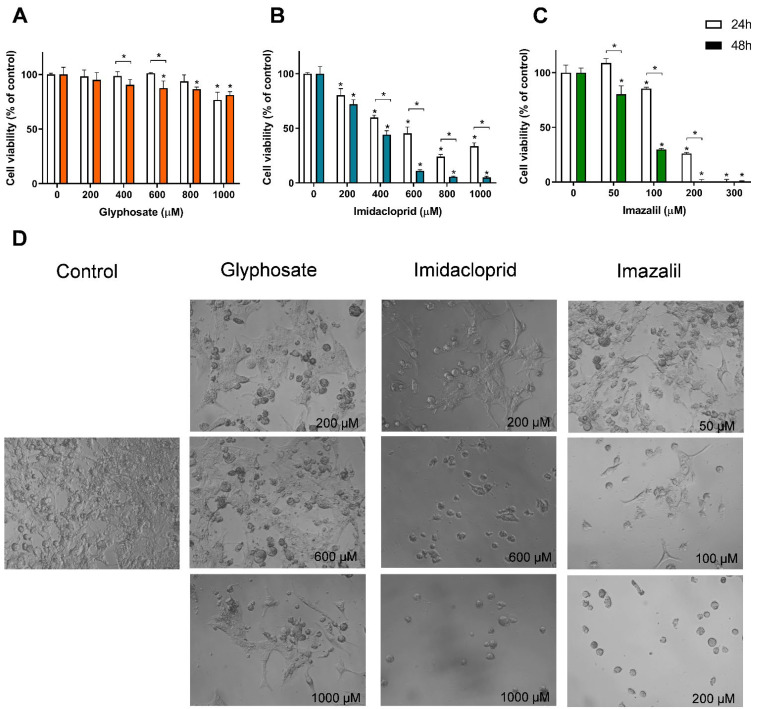
Effect of pesticides on SK-MEL-5 cells viability and on cell morphology. SK-MEL-5 cells were exposed to GLY (**A**), IMD (**B**) and IMZ (**C**), for 24 h and 48 h. Cell viability was assessed using Alamar Blue assay, and bright-field microscopy was used to assess pesticide-induced morphological changes (**D**). Significant statistical differences between the control and samples are denoted as “*” and between exposure times for the same concentrations are denoted as “*” over a bracket, when *p* < 0.05.

On the other hand, the macrophages were the most affected cell line when exposed to IMD and IMZ (Table 1). GLY was not able to induce cell death below 50% in any of the tested cell lines, even when applied at 1 mM (highest tested concentration). SK-MEL-5 cells were the only cell line that presented a significant reduction in cell viability when exposed to 1 mM of GLY, presenting a reduction of 23.3% and 18.7% in cell viability at 24 h and 48 h, respectively (Figure 3A). Among the human skin cell line models, HaCaT and SK-MEL-5 cells presented similar IC_50_ values at 24 h, while A431 cells were less sensitive to IMD toxicity. At 48 h, HaCaT cells depicted the highest cell viability, where the increase in exposure time did not significantly affect the cytotoxicity (*p* > 0.05). Concerning IMZ, HaCaT is also the skin cell line less affected by the fungicide-induced toxicity at both exposure times (Table 1). The dose-response effect and the morphological alteration of SK-MEL-5 cells exposed to the three pesticides are shown in Figure 3, as this cell line presents a distinct morphology and the lowest IC_50_ values at 48 h exposure to both IMD and IMZ (comparison between skin cell lines).

Unlike what was observed for Caco-2 cells, in melanocytes exposed for 48 h to 1 mM of GLY, reduction in cell density was seen, together with changes in cell morphology (Figure 3D), which is in line with the observed reduction in cell viability (Figure 3A). Control SK-MEL-5 cells present a polyhedral shape with many membrane projections (pseudopodia-like structures), but in the presence of GLY the number of round cells augmented (Figure 3D). As seen in Figure 3B, IMD induced a reduction of ~88% in SK-MEL-5 cells’ viability, at 600 µM (48 h exposure) and, as seen in Figure 3D, only a small population of cells presenting a round shape remained, without the typical plasmatic membrane ramifications. Even at 200 µM, IMD induced morphological changes by decreasing cells’ density and the plasmatic membrane ramification; which is in line with the decreased cell viability (Figure 3B).

Concerning the effect of IMZ in SK-MEL-5 cells, the same pattern was observed (Figure 3D), i.e., a dose- and time-dependent cytotoxicity (Figure 3C) as well as changes in cell morphology into round-shaped cells (Figure 3D). As observed (Figure 3C), concentrations as low as 50 µM, after 48 h exposure, reduced cell viability in about 20% (cell viability of ~80%; Figure 3C). The IMZ IC_50_ at 48 h exposure was 76.7 ± 1.8 μM, a value that is higher than that observed for HepG2 cells (47.1 ± 0.5 μM) and RAW 264.7 cells (7.21 ± 4.5 μM), and lower that that observed for the other cell lines (Table 1), denoting a moderate sensibility to IMZ, compared with the studied cell lines.

Hepatocytes, here modeled by HepG2 cells, are specialized in the metabolism of xenobiotics, where toxic molecules are metabolized into a series of derivatives aiming to reduce toxicity and to increase the excretion rate [29,30]. HepG2 cells were not affected by GLY exposure in concentrations up to 1 mM (Table 1), and showed the second highest IC_50_ value for IMD exposure, below Caco-2 cells only (Table 1), which presupposes a significant resistance of both the intestinal tract cells and hepatocytes to this insecticide. Nevertheless, HepG2 cells are greatly affected by IMZ exposure, where a significant reduction in cell viability was observed at 50 µM. HepG2 cells presented the second lowest IC_50_ value for IMZ-induced toxicity; RAW 264.7 cells exhibited higher sensitivity to this fungicide (Table 1).

## 4. Discussion

In the present research, a comparison between the toxicity of three widely used pesticides, namely an herbicide, an insecticide and a fungicide, was carried out in various animal cell line models of different organs/tissues.

A previous study in Caco-2 cells showed that GLY concentrations higher than 10 mg/mL (~59 mM) disrupted Caco-2 monolayers, interacting with the actin cytoskeleton and also inducing loss of membrane integrity (observed as lactate dehydrogenase leakage) [31]. A second study, using a GLY-based formulation, reported an IC_50_ value in Caco-2 cells of 17.2 mg/mL (~102 mM), and also reported the effect of the adjuvants (such as surfactants) present in GLY-based herbicides as responsible for much of the toxicity, as they presented much higher toxicity [19]. However, these are concentrations 59-fold, and 102-fold higher than the highest concentration tested in our study, as our approach was aimed at concentrations closer to the real exposure, as reported in the literature. In human serum, after accidental exposure GLY was found to be present at 89 µg/mL (~0.52 mM) [1]. Considering food exposure for a person with a healthy US-style diet, the worst-case scenario of GLY exposure was reported to range between 2954 µg/day (i.e., 17.47 µmol/day) and 3142 µg/day (18.59 µmol/day) [32]. In a recent study concerning glyphosate occurrence in food products, cereals were the foods with higher content [33], with a sample of wheat seeds (from Italy) having the higher content (230 mg/kg, i.e., 1.36 mmol) [33,34]. Thus, as mentioned above, most studies used much higher concentrations of GLY than the real exposure. Here we have selected a range of concentrations up to 1 mM, which is more realistic when considering the concentration and accumulation of daily diet exposure. It has also been reported that glyphosate is not accumulated in the body, and is eliminated in about two weeks through the feces and urine; in a survey study the highest value found in urine was of 130 μg/L (<1 μM) [35]. Concerning IMZ, in post-harvest citrus fruit, concentrations up to 10 mg/kg were found in lemon peel [36] (i.e., 0.034 mmol/kg). Regarding IMD, a study performed in India reported the presence of this insecticide in many food products and estimated a daily intake of IMD of 0.004–0.131 µg/kg body weight [37]. Neonicotinoids, including IMD, were detected at concentrations of 0.24 to 57.3 ng/L in drinking water samples [38]. Due to the insufficient data available regarding the quantity of IMD and IMZ in food, and thus their intake through diet and bioaccumulation, we have used the same range of concentrations of all pesticides to assess their toxicity and to compare the data. Concerning IMD, Caco-2 cells exposed for 96 h to concentrations higher than 0.25 µg/mL (~0.98 μM) showed a reduction in cell viability (81% of cell viability 0.25 µg/mL) [21], but the results here presented (Figure 2 and Table 1), with a shorter incubation (24 or 48 h), showed a reduction in Caco-2 cell viability only for concentrations higher than 200 µM. However, we have used the IMD standard and not a formulation of the pesticide. Nedzvetsky et al. [21] reported the source as a formulation from Bayer, that contains other compounds such as emulsifiers, surfactants and other substances that are highly toxic [1]. Tao et al. [20] reported an IC_50_ = 30 µg/mL (~100 µM) for Caco-2 cells exposed to IMZ during 24 h, a value that is 2.53-fold lower than the IC_50_ here reported (Table 1). While our methodology uses Caco-2 cells seeded at 5 × 10^4^ cells/mL (100 μL/well) and allowed to adhere for 48 h, Tao et al. [20] used a cell density of 5 × 10^3^ cells/well but only 24 h of adhesion period; they also used a different methodology to determine cell viability, which may explain the different IC_50_ together with the fact that their cell viability reduction at 60 µg/mL (~200 µM) is identical to that induced by 30 µg/mL; thus, the findings might not be so different from ours. Nevertheless, the authors also observed morphological changes of Caco-2 cells exposed to IMZ [20], identical to those we report here. In HepG2 cells exposed to low concentrations of GLY (up to 3.5 µg/mL; i.e., 20.7 µM), a slight increase in cell proliferation at 4 h and 24 h exposure was reported [22]. Concerning the effect of GLY in skin cells, in a melanocyte cell line (SK-MEL-2), an IC_50_ of 11 µM after 72 h exposure to GLY was reported [39], while in HaCaT cells, Heu et al. [40] reported IC_50_ of 30 mM after 18 h exposure [40]. To the best of our knowledge, there are no studies concerning the effect of GLY and IMD on RAW 264.7 and on A431 cells, the ones reported here being a novelty (Table 1).

Singh et al. [41] recently reported that IMD at 2.35 mM reduces HaCaT cells viability below 50%, when exposed for 24 h. This value is 4.74-fold higher than the one reported in our research (Table 1), which can be explained by the difference in cell density per well. Regarding HepG2, Guimarães et al. [42] reported that IMD dose-dependently reduced cell viability in the range of 0.5–2.0 mM or 0.25–2.0 mM, for 24 h or 48 h exposure, respectively, values well aligned with the IMD toxicity against HepG2 cells (Table 1).

Concerning IMZ toxicity against cell lines, there are a few studies published, almost limited to Caco-2 cells. Thus, here we here report for the first time, to the best of our knowledge, the comparison between new cell line models (not previously studied) with relevance for IMZ-induced toxicity, given the routes of contact with the fungicide.

As these cell lines do not have the relevant molecular targets for these pesticides, and as we observed in Table 1, there is a general rank of toxicity GLY < IMD < IMZ that seems to be independent on the cellular specific proteins (i.e., receptors, transporters, etc.). We hypothesize that pesticides’ intrinsic molecular characteristics might determine the observed toxicity.

**Figure 4:**
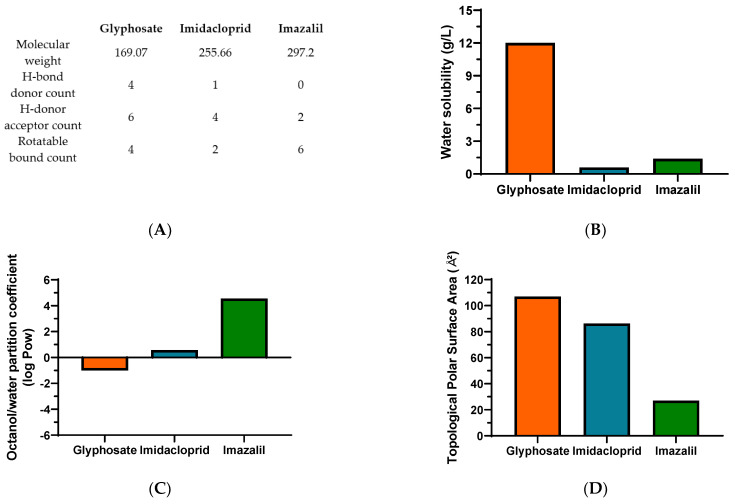
Physicochemical properties of glyphosate, imidacloprid and imazalil relevant to their toxicity to human cell line models. Pesticides’ molecular weight and relevant chemical bonds information (**A**), water solubility (**B**), octanol/water partition coefficient (**C**) and topological surface area (**D**). Data was obtained from Pubchem and INCHEM (IPCS, World Health Organization) [49,50,51,52].

In Figure 4, relevant physicochemical properties of the selected pesticides which we consider relevant for their toxicity, are summarized. The toxicity of a xenobiotic is highly dependent on defined characteristics that facilitate its interaction with the cell membranes and its ability to permeate them. Various rules have been described and applied in drug development, aiming to predict the potential of a drug to target the cell, which may also provide an insight about the toxicity of pesticides. Lipinski et al. [43] described a “rule of 5”, where key physicochemical properties are likely to induce poor oral absorption or permeation of a drug: molecular weight above 500, Log P_ow_ higher than 5 and hydrogen-bond (H-bond) donors and acceptors higher than 5 and 10, respectively. The authors also reported that molecules, such as biological transporters with affinity to these drugs as substrates, induced exceptions to the rule [43]. Later, Lipinski [44] and Veber et al. [45] introduced updates to the rule, adding the count of rotatable bonds (>10), linked with higher permeability through barriers (e.g., intestinal barrier), since the increase in rotatable bounds decreased ligand affinity [44]. The effect of polar surface area (PSA; ideally less than 140 Å^2^) and the affinity for xenobiotic efflux pumps were also addressed. It was noted that PSA is directly correlated with permeation, with higher significance than lipophilicity [44,45]. Additional attempts to select potential drug candidates in the early stages was improved with the development of methods such as quantitative estimate of drug-likeness and quantitative structure-activity relationships (QSAR), aiming to provide a more accurate classification of the drug potential based on the interactions of the various criteria instead of an exclusive set of rules [46,47,48]. Nevertheless, these rules were mainly defined for oral intake, not considering other administration routes. Considering the data here presented, we attempted a correlation with these physicochemical characteristics, aiming to evaluate the potential to permeate and bioaccumulate in different in vitro cell models originated from different tissues.

Starting with the molecular weight (MW), all molecules present a MW lower than 500 (Figure 4A). An inverted trend is seen between the IC_50_ values shown in Table 1 and the MW, as all cell lines presented higher sensitivity, and thus lower IC_50_ values, to IMZ, the compound with higher MW.

**Figure 5:**
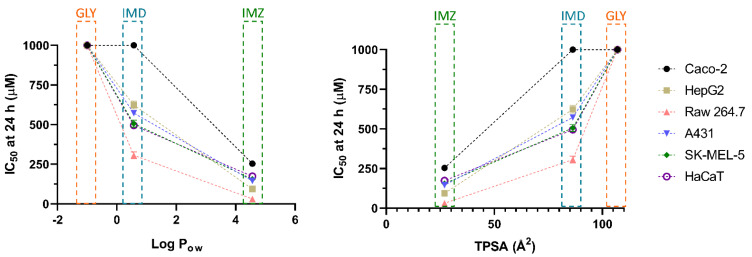
Correlation between Log P_ow_ (**left**) and TPSA (**right**) with the IC_50_ values obtained from in vitro cytotoxicity assays (see Table 1), for cells exposed for 24 h.

Concerning membrane permeation, xenobiotics can follow various routes, either passively (moving down the concentration gradient, as paracellular or transcellular transport) or actively. In the first case, lipophilicity is a critical characteristic of xenobiotics, determining the ability to cross or be retained in the lipid bilayer barrier. The partition coefficient in an *n*-octanol/water system (log P_ow_) is a common assay to measure the hydrophilicity or lipophilicity of a substance [53,54]. An extensive review of drug absorption using Caco-2 cells, and formulated with QSAR, demonstrated the usefulness of log P_ow_ in the assessment of a compound’s permeation and absorption. An average value of optimum log P_ow_ was 2.94 [48]. Regarding the pesticides under analysis in the present research, for GLY, IMD and IMZ the reported log P_ow_ values are: −1.0, 0.57 and 4.56, respectively (Figure 4C). Thus, IMZ presents the highest log P_ow_ and the closest to the ideal average value of 2.94 reported by Hansch et al. [48]. Thus, GLY presenting a negative log P_ow_, is hydrophilic, as also shown by its water solubility (Figure 4B), which is 20-fold and 8.57-fold higher than IMD and IMZ solubility in water, respectively. In fact, GLY has high polarity and it also presents very low solubility in organic solvents [55]. Therefore, the polar molecule of GLY presents higher affinity to the aqueous phase (culture media), while IMD and IMZ present higher affinity to the lipophilic phase (cell membranes). Within the results reported here, the log P_ow_ correlates directly with the observed toxicity, as IMZ presents the lowest IC_50_ values for all tested cell lines (Table 1 and Figure 5).

The polar surface area has also been considered a relevant physicochemical property for the prediction of a molecule’s permeation/absorption. Briefly, it represents the sum of oxygen, nitrogen and their linked hydrogen atoms surface area, which are main functional groups. To ease the application of this parameter, more recent studies use the topological polar surface area (TPSA) index, proposing that molecules with TPSA > 140 Å^2^ present low membrane permeation, while compounds with TPSA < 60 Å^2^ have higher ability to permeate biological membranes [56,57]. Besides, MRP1 (multidrug resistance associated protein 1), as xenobiotics’ efflux pump, has been linked to TPSA. It has been proposed that molecules with higher TPSA are substrates of MRP1, and their conjugation with glutathione (GSH), such as conjugation of GSH and xenobiotics, is a standard mechanism to increase their efflux. This may increase the molecule’s TPSA, and thus increase its transport, when compared to its unconjugated form with lower TPSA [57,58]. As observed for log P_ow_, the TPSA perfectly aligns with the observed toxicity, as GLY presents the highest value (107 Å^2^), while IMD (86.3 Å^2^) and IMZ (27 Å^2^), present progressively lower TPSA and lower IC_50_ values (Figure 4D and Figure 5). Similar findings were proposed for P-gp (P-glycoprotein), another ABC efflux transporter, where a TPSA < 60 Å^2^ was a contributing factor to defining molecules as non-P-gp substrate [59]. Combining both the log P_ow_ and TPSA values, it is reported that molecules simultaneously presenting low log P_ow_ and high TPSA tend to be more easily removed and be less toxic, while the opposite ratio (high log P_ow_/low TPSA), increased the molecule’s toxicity [60].

As mentioned above, in Lipinski et al. [43], the “rule of 5”, together with hydrogen-bond (H-bond) donors higher than 5 and H-bond acceptors higher than 10, indicates low membrane permeability. Among the three compounds under study, all present H-bond acceptor counts lower than 10, with the highest count being observed for the polar compound GLY, and the count decreasing as GLY > IMD > IMZ. The same tendency was observed from the H-bound donor count, where IMZ presents zero hydrogen-bond donors (Figure 4A). These results follow the correlation observed for log P_ow_ and TPSA regarding the cytotoxicity of the various cell lines tested. It has been shown that strategies aiming to reduce/eliminate H-bond acceptors and donors improve membrane permeation, and it was also observed that lower H-bond acceptor counts reduce the affinity as substrate for P-gp efflux pump [59,61]. In addition to H-bond count, the rotatable bonds count helps to predict passive membrane permeation. The rigidity of a molecule is thought to promote the permeabilization, in opposite to molecule flexibility, where a count of less than 10 rotatable bounds is an indicator of potential higher permeation [62]. Although all our compounds fit within this range, in contrast with the physicochemical properties described above, IMZ presents the highest rotatable bound count, suggesting that it represents a less prominent factor considering the in vitro analysis here performed and when compared to GLY and IMD. In addition to the above-mentioned parameters, other physicochemical properties may also play a role in xenobiotics’ permeation/absorption, such as their acidity/alkalinity, reported as the pKa value(s) [63]. This parameter correlates with the molecule ionization status and is a key property that modulates permeation/absorption. In order to permeate biological barriers, the presence of a charge at physiological pH is known to reduce membrane permeation, when compared to non-charged molecules [64]. It was reported that compounds with at least one charge and pKa < 4 or pKa > 10, for acids and bases, respectively, were not able to permeate the blood-brain barrier, and molecules with these pKa values will assume the charged conformation at physiological pH [65]. On the other hand, molecules in their non-ionized form present increased lipophilicity and are more likely to diffuse through biological membranes [63]. Physiological pH is highly dependent on the considered tissue; it can range between 6.5–7.4 in the intestinal barrier (small intestine lumen, enterocytes and blood vessels network), with a broader range between 1.7–8 when considering the gastrointestinal tract, while plasma has a tightly controlled pH value of 7.4 [66]. Thus, at a reference physiological pH of 7, molecules with pKa in the range of 6–8 present an equilibrium, where at least 10% is ionized [66]. Among the pesticides tested in the present research, IMZ is a weak acid, with a pKa = 6.53 [49], the closest to physiological pH. GLY is a weak acid with four ionizable forms (pKa values of 2.0, 2.6, 5.6 and 10.6) [50]. Therefore, when comparing both compounds in culture media (pH 7–7.5), GLY will have on average ~2 negative charges being repelled by the negative charges of the cellular lipid bilayer, disfavoring the GLY-lipid interaction and the possibility of diffusion through the bilayer, when compared to IMZ which at physiological pH has a higher percentage of molecules in a non-ionized form. IMD presents two ionization positions with pKa values of 1.56 and 11.12, and therefore is likely to present a higher percentage of its charged (cation) status in pH values ranging between 5 and 9 [51]. Thus, at physiological pH, IMD and GLY should have lower capacity to permeate biological barriers, when compared to IMZ, which is in line with the observed IC_50_ values, where IMZ presents the highest toxicity and also correlates with the lipophilicity and log P_ow_ reported in Figure 4. Comparing IMD and GLY, IMD is more likely to interact with cellular membranes than GLY, which also correlates with the reported toxicity.

The influx and efflux of xenobiotics by transporters present in the cells’ plasmatic membrane also play a significant role in pesticide-induced cytotoxicity. As discussed above, molecules with lower TPSA have less affinity as MRP1 substrate [57]. P-glycoprotein (P-gp), one of the most relevant members of the ATP-binding cassette family (ABC), has also been studied for its role in pesticide efflux. It has been hypothesized that molecules with lower H-bond count and low TPSA rapidly and passively permeate cell membranes. As P-gp transporters can bind xenobiotics in the cytosolic side of plasmatic membrane, these molecules can escape P-gp more easily [59]. This may be the case for IMZ.

Using Caco-2 cells exposed to GLY, Xu et al. [67] observed that GLY uptake was ATP-independent, and that L-system transporters 1 and 2 (LAT1 and LAT2) were shown to actively participate in GLY influx in Caco-2 cells, as its physicochemical properties are not compatible with large passive diffusion. In a study using brown planthopper as experimental model, it was shown that an ABC transporter coded by the gene *NlMdr49-like* is involved in IMD resistance [68], showing that ABC efflux pumps may be significant assets in protecting from IMD-induced cell damage through their efflux activity and thus reducing bioaccumulation. A different study using Caco-2 cells reported that IMD uptake can occur both passively or via transporters, but its efflux although regulated by an efflux pump, was not likely to be connected to P-gp or MDR proteins [69]. IMZ has been shown to inhibit CYP3A4, a key member of mammalian cytochrome P450 involved in xenobiotics’ metabolization [70], being both a hazard when considering co-ingestion with drugs as it modulates their bioavailability, but also potentially reducing its own degradation.

Thus, the variations between the cytotoxicity induced by the three tested pesticides are highly correlated with physicochemical parameters such as the log P_ow_, TPSA and the H-bond count. Accordingly, IMZ presents the highest cytotoxicity against all the tested cell lines, as it presents physicochemical characteristics that potentiate a greater and faster passive diffusion, while GLY as a polar molecule and with low lipophilicity is less likely to interact with the lipid bilayer and accumulate intracellularly at toxic concentrations.

Regarding the increased toxicity between cell lines exposed to the same pesticide, Caco-2 cells, the model for the intestinal barrier, present a lower sensitivity to pesticide exposure. The intestinal barrier is a primary contact point between the organism and ingested xenobiotics, and these cells have a large number of mechanisms for xenobiotic efflux and metabolization, and are likely more capable of countering pesticide-induced toxicity. Similarly, hepatocytes are specialized in toxics’ metabolism, and it is thus no surprise that they also present a lower sensitivity to IMD. Nevertheless, IMZ induced significant toxicity to HepG2, requiring a further analysis of the mechanism of action underlying a specific hepatic toxicity of the fungicide. RAW 264.7 cells were observed as the most sensitive to IMD and IMZ toxicity. Unlike hepatocytes, whose primary activity is xenobiotic metabolism, or cells from the intestinal or skin barriers, macrophages are less likely to exhibit morphological adaptations to xenobiotics. Macrophages present a distinct expression of membrane transporters, with low expression of P-gp, but high expression of MRP1 and MRP3, organic anions transporters and monocarboxylate transporter [71,72]. In addition to a different expression profile for membrane transporters, macrophages exhibit the ability to phagocyte exogenous substances [73], which may increase their uptake of these later. These factors can contribute to an increase in IMD- and IMZ-induced toxicity.

Therefore, pesticide-induced toxicity can be correlated to the chemical’s physicochemical properties, such as MW, pKa, H-bonds (donor and acceptor), solubility, TPSA and log Pow. Compounds simultaneously presenting low TPSA, low H-bond count and high TPSA are likely to induce significant toxicity in skin and intestinal tract tissues, as these characteristics are strongly correlated with an increased passive diffusion through biological barriers. Thus, the analysis of these key physicochemical parameters may allow the construction of a predictive model for addressing potential exposure to common pesticides and other xenobiotics. This study proposes the basis for a predictive model, that should be extended to a greater number of pesticides and other xenobiotics to further validate its effectiveness.

## 5. Conclusions

Pesticides-induced toxicity has been the target of a high number of published studies in various in vitro and in vivo models, aiming to ascertain their safety levels for both general population and occupation hazard. In this study, we present the comparison of a common herbicide (GLY), an insecticide (IMD), and a fungicide (IMZ) induced toxicity in various animal cell lines originated from different tissues. Comparing the pesticides toxicity, GLY was the least toxic, as it did not reduce cell viability or alter cell morphology, which can be explained by the fact that GLY-target enzyme is not present in animal cell lines, thus not interfering with cell metabolism, but also because the molecule is highly hydrophilic which reduces the capacity of GLY to interact with cell membranes. However, we may not exclude the possibility that, in specific cell lines, GLY is taken up by cells and induces intracellular deleterious events such as oxidative stress, which should be analysed in future research. Considering the molecular targets of IMZ and of IMD, IMZ hampers the cytochrome P450-dependent demethylation of lanosterol while IMD targets nAChRs. While the cytochromes P450 superfamily of enzymes, which in mammals are mainly found in liver and gut (here mimicked by HepG2 and Caco-2 cells), are responsible for the metabolism of xenobiotics; the nAChRs are mainly found in muscle and neuronal cells (none of these were here studied). Considering the toxic effect of IMZ on HepG2 cells, its effect on xenobiotic metabolism should not be excluded and should be further analysed. In addition, the action of pesticides on P450 enzyme system may interfere with the rate of ATP synthesis and thus with the rate of oxygen consumption, which could lead to increase in oxidative stress and this also should be further analysed. In this work, we observed that intestinal barrier cells are the more resistant to the pesticide’s exposure, which is correlated to their high capacity to perform the efflux of xenobiotics, in contrast to macrophages, suggesting that systemic circulation of IMD and IMZ may induce significant toxicity. Besides these potential sites of toxicity, due to IMD and IMZ hydrophobic nature, the interaction of these two pesticides with plasmatic membrane components may lead to changes in normal components (e.g., transporters, channels, enzymes) activity which may result in oxidative stress and/or induction of apoptosis which deserves future attention. In addition, physicochemical factors such as *n*-octanol/water partition coefficient, TPSA and H-bond are likely to be directly correlated with pesticide-induced toxicity, thus being key factors to potentially predict the toxicity of other compounds.

## Figures and Tables

**Table 1:** IC_50_ values (in µM) of the tested cell lines exposed to glyphosate, imidacloprid and imazalil.

Cell Line	Exposure Time	Glyphosate		Imidacloprid		Imazalil	
A431	24 h	>1000	n.s.	573.2 ± 10.4	*	145.0 ± 1.7	*
	48 h	>1000		389.3 ± 9.2		117.1 ± 6.5	
HaCaT	24 h	>1000	n.s.	495.3 ± 19.4	n.s.	173.6 ± 15.3	*
	48 h	>1000		482.3 ± 12.9		120.2 ± 6.2	
SK-MEL-5	24 h	>1000	n.s.	506.6 ± 22.07	*	155.7 ± 9.0	*
	48 h	>1000		318.8 ± 11.5		76.7 ± 1.8	
Caco-2	24 h	>1000	n.s.	>1000	*	253.5 ± 3.37	*
	48 h	>1000		832.1 ± 29.6		186.5 ± 2.27	
HepG2	24 h	>1000	n.s.	623.8 ± 24.3	n.s.	93.7 ± 2.2	*
	48 h	>1000		620.2 ± 10.6		47.1 ± 0.5	
RAW 264.7	24 h	>1000	n.s.	305.9 ± 22.4	n.s.	31.3 ± 2.7	*
	48 h	>1000		306.6 ± 22.1		7.21 ± 4.5	

Notes: Results are presented as mean ± S.D.; n.s.—not significant; Significant statistical differences between exposures times were denoted as “*” when *p* < 0.05.

## Data Availability

Not applicable.
